# Cost-Effective Production of Bacterial Cellulose and Tubular Materials by Cultivating *Komagataeibacter sucrofermentans* B-11267 on a Molasses Medium

**DOI:** 10.3390/polym17020179

**Published:** 2025-01-14

**Authors:** Marina V. Parchaykina, Elena V. Liyaskina, Alena O. Bogatyreva, Mikhail A. Baykov, Diana S. Gotina, Nikita E. Arzhanov, Alexander I. Netrusov, Viktor V. Revin

**Affiliations:** 1 Department of Biotechnology, Biochemistry and Bioengineering, National Research Ogarev Mordovia State University, 430005 Saransk, Russia; liyaskina@yandex.ru (E.V.L.); bogatyrevaao@mail.ru (A.O.B.); baikoffworkmail@gmail.com (M.A.B.); gdiana23@mail.ru (D.S.G.); snakenfive@yandex.ru (N.E.A.); revinvv2010@yandex.ru (V.V.R.); 2 Department of Microbiology, Faculty of Biology, M.V. Lomonosov Moscow State University, 119234 Moscow, Russia; anetrusov@mail.ru

**Keywords:** bacterial cellulose, molasses, biopolymers, functional materials, tubular structures, biocomposites, biomedicine

## Abstract

An original design of a simple bioreactor was used to fabricate two tubular, 200 cm long BC structures by culturing *Komagataeibacter sucrofermentans* B-11267 on a molasses medium. In addition, a tubular BC-based biocomposite with improved mechanical properties was obtained by combining cultivation on the molasses medium with in situ chemical modification by polyvinyl alcohol (PVA). Moreover, the present study investigated the BC production by the *K. sucrofermentans* B-11267 strain on the media with different molasses concentrations under agitated culture conditions. The dynamics of sugar consumption during the cultivation were studied by HPLC. The structure and physicochemical properties of BC and tubular BC structures were characterized by FTIR spectroscopy and X-ray diffraction (XRD). Thus, the findings indicate that *K. sucrofermentans* B-11267, when cultivated in a molasses medium, which is such a cheap waste product in the sugar industry, forms a significant amount of BC with a high crystallinity degree. The BC tubular structures demonstrated great potential for their application in biomedicine as artificial blood vessels and conduits for nerve regeneration.

## 1. Introduction

**Figure 1:**
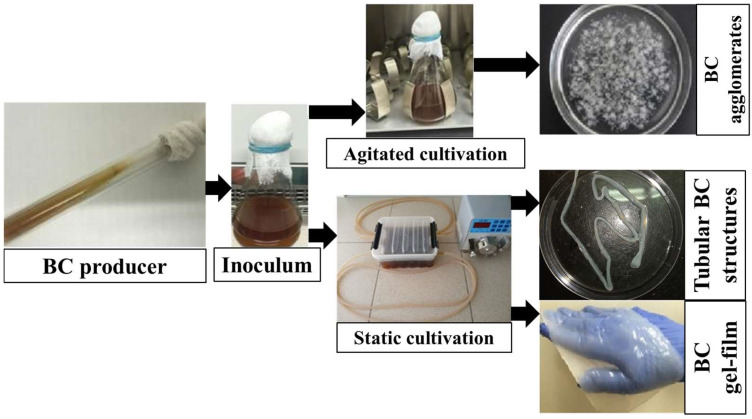
Scheme of BC production and tubular materials by cultivating *K. sucrofermentans* B-11267 on a molasses medium.

Recently, bacterial cellulose (BC) has attracted the attention of scientists around the world as a biopolymer with unique beneficial properties with wide possibilities of application in various fields, including biomedicine, nanoelectronics, food industry, environmental remediation, etc., [1,2,3,4,5,6,7,8]. Due to the unique properties of BC and its great prospects for use in various fields, particularly in medicine, the issue of cost-effective BC production is currently critical. The main strategies in creating such production are, first of all, the development of cheap nutrient media, the search for highly productive strains of microorganisms, and the optimization of their cultivation conditions [2,9,10,11,12,13]. Thus, many researchers have proposed various industrial and agro-industrial strategies utilizing food waste, including dairy, alcohol, and sugar industry by-products, for cost-effective BC production [14,15,16,17,18,19,20]. A huge amount of molasses is produced worldwide every year, making it an easily accessible resource for cellulose production. Molasses is a by-product of processing sugar cane and sugar beet in sugar production. The cost of molasses is only 1/250 of that of fructose [19], but it is very rich in minerals, organic compounds, and vitamins. Machado et al. (2018) showed the ability of sugarcane molasses to contribute to a significant reduction in the cost of media (up to 20.06%) [20]. BC production is carried out by aerobic fermentation, and unlike other microbial exopolysaccharides, not only under agitated culture conditions but also under static conditions (Figure 1).

In this case, the BC gel film is formed on the medium surface. Under agitated culture conditions, BC agglomerates of various shapes and sizes are formed depending on the strain, medium, and cultivation conditions used [9]. BC production in static culture is simpler, and requires little technological investment, but it is based on the use of large nutrient medium surfaces to overcome oxygen diffusion limitations, and usually requires a long fermentation time [9]. When culturing producers under dynamic conditions, strong mechanical mixing prevents heterogeneity in the nutrient medium and promotes the oxygen saturation of the medium, while control and scaling can be implemented more easily [9]. Therefore, the method is preferable for industrial production of BC. However, it is more energy-intensive, and can contribute to the emergence of mutants that do not produce cellulose [9]. BC gel films have been used in medicine as wound dressings for a long time, and have prospects to be used in tissue engineering, nanoelectronics, ecology, etc. BC particles obtained in agitated culture have proven useful for immobilizing enzymes, creating injectable orthopedic scaffolds, incorporating drugs, and removing dyes and heavy metal ions [10,21,22]. In terms of strain productivity under static and agitated cultivation conditions, the data in the literature are contradictory. A number of scientists reported a higher yield of BC under static cultivation [13], while other researchers, on the contrary, reported this under dynamic cultivation [14]. However, such a comparison is not always reasonable without taking into account the time and conditions of producer cultivation. In terms of the physical and mechanical properties of BC, most scientists agree that biopolymers obtained under static culture conditions have a higher degree of crystallinity, higher tensile strength, a denser network structure, and higher thermal stability, while BC obtained under stirred culture conditions have larger pores and a lower degree of crystallinity [23,24].

Since BC is a promising material for medicinal applications, various functional materials in the form of films, hydrogels, and aerogels have been developed on this basis [4]. A new, interesting material for medical use is tubular structures that can be used to create artificial blood vessels and conduits for nerve regeneration. Such materials should have sufficient strength, elasticity, and biocompatibility [25,26]. BC, due to its unique properties, is an ideal candidate for obtaining these materials [26,27]. However, some properties of BC tubular structures, including mechanical characteristics and elasticity, do not fully meet the necessary requirements for clinical use, and require BC modification [28,29]. The problem can be solved by obtaining BC-based biocomposites using some natural or synthetic polymers, such as gelatin, collagen, polyvinyl alcohol (PVA), etc. For example, Bao et al. (2022) developed a tubular composite with BC and fish gelatin with improved mechanical properties for application as a small-diameter artificial blood vessel [30]. In another study, Tang et al. (2015) obtained tubular BC/PVA composites with a significantly greater tensile strength and water permeability by using a thermally induced phase separation method [31].

Recent studies have shown that the structures and properties of BC tubes can be regulated by cultivation conditions including nutrient media, bacterial strain type, oxygen ratio, incubation time, and cultivation bioreactor [32]. An interesting review by Roberts et al. (2023) summarized the strategies used to fabricate tubular BC structures, and reported various types of bioreactors, each with different advantages and disadvantages, yielding BC tubes with varying properties [32]. Therefore, the authors concluded that there is still a great need for further research within this area. It should be noted that the known tubular BC structures were obtained in the media, which contain expensive components, e.g., glucose, fructose, peptone, yeast extract. As far as we know, molasses has not been used to obtain tubular BC structures, although it is widely used for BC production. Molasses does not only reduce the cost of media, but also promotes the formation of a biopolymer with high mechanical strength and high crystallinity [11,18].

The aim of the present work was to study BC production and tubular BC structures under specific cultivation conditions on a molasses medium. In addition, a tubular BC-based biocomposite was obtained by combining the cultivation on the molasses medium with in situ chemical modification by PVA to improve its mechanical properties.

## 2. Materials and Methods

### 2.1. BC Production

In this study, *Komagataeibacter sucrofermentans* B-11267 was used in the production of BC. The *K. sucrofermentans* B-11267 strain was isolated from Kombucha tea and deposited in the Russian National Collection of Industrial Microorganisms (VKPM) (accession no.: B-11267) [33]. This strain was cultured in media with beet molasses concentrations of 50 g/L (M_50_), 70 g/L (M_70_), 90 g/L (M_90_), and 110 g/L (M_110_), with a pH 4.5, and Hestrin and Schramm (HS) medium (g/L): glucose (20) (OOO Promsintez, CAS No. 5996-10-1, Chapayevsk City, Russia, extra pure), peptone (5) (Research Center for Pharmacotherapy, Saint Petersburg, Russia), yeast extract (5) (State Research Center for Applied Microbiology and Biotechnology, Obolensk, Russia), citric acid (1.15) (chemical reagent, Nizhny Novgorod, Russia, CAS number: 77-92-9, extra pure, purity 99.8%), and disodium hydrogen phosphate (2.7) (LabTech, Moscow, Russia, CAS № 7783-28-0, Pro Analysi), pH 6.0. The sugar beet molasses used in this study were obtained from the Romodanovsky Sugar Factory (Mordovia, Russia). The beet molasses had the following composition (% by weight): dry matter 82.0; sucrose 50.0; total nitrogen 2.3. The media were autoclaved at 121 °C for 20 min prior to inoculation.

The inoculum for seeding nutrient media was prepared using a method we have previously described [18]. BC was produced under agitated culture conditions at 28 °C for 6 days at 250 rpm. After incubation, BC was collected, washed thoroughly with deionized water to remove medium components, and treated with 1% (*w*/*v*) sodium hydroxide solution (LabTech, Moscow, Russia, CAS № 1310-73-2, extra pure) for 1 h at 80 °C to eliminate bacterial cells. Furthermore, BC was rinsed extensively with 6% (*v*/*v*) acetic acid (Chem Med, Moscow, Russia, CAS № 64-19-7, extra pure) and then deionized water until the pH became neutral. The purified BC was dried to a constant weight at 60 °C. BC production is reported as gram dry weight of cellulose per liter of medium (g/L).

### 2.2. Production of BC and BC/PVA Tubular Structures

**Figure 2:**
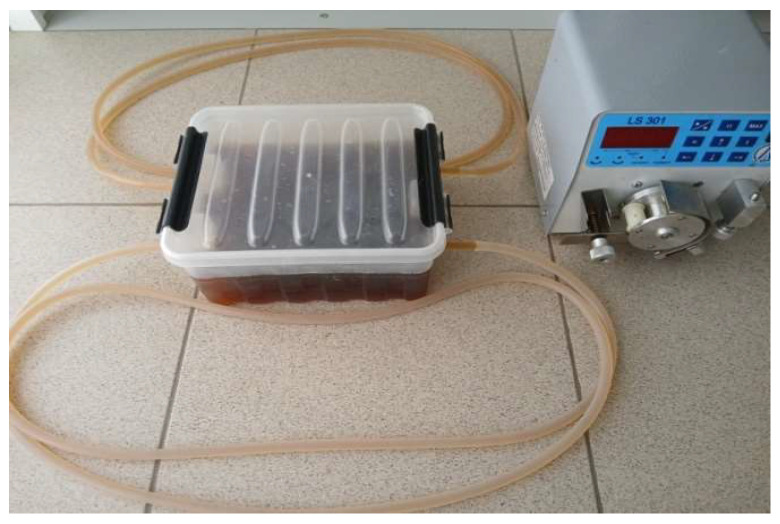
Container with two silicone tubes for producing BC tubes.

BC tubular structures were produced by fermenting *K. sucrofermentans* B-11267 in a container with two 200 cm silicone tubes, their internal diameter being 5 mm, using a medium with molasses concentrations of 50 g/L (M_50_) (Figure 2). The medium was inoculated with 10% (*v*/*v*) inoculums obtained by the method described previously [18]. For this purpose, 1350 mL of liquid nutrient medium and 150 mL of inoculum were added to the container under aseptic conditions. Each silicone tube contained 39 mL of culture medium. The *K. sucrofermentans* B-11267 was cultured for 7 days with double mixing of the nutrient medium in each tube for 10 min on days 1 and 2. A LOIP LS-301 peristaltic dosing pump (LOIP, St. Petersburg, Russia) at a rotor speed of 200 rpm was used for mixing. The liquid flow rate was 53 L/h. At the end of the process, the nutrient medium and the resulting gel film were removed from the container, with 1500 mL of distilled water added. To release the tubular structures from the silicone tubes, a process similar to mixing the medium was carried out, during which the biopolymer was pushed out of the tubes by a stream of water. The resulting structures were processed to remove cells and components of the nutrient medium by the method described previously [18]. Finally, the BC tubular structures were autoclaved at 121 °C for 20 min. Composite tubular structures based on BC and polyvinyl alcohol (PVA) (LabTech, Moscow, Russia, CAS: 9002-89-5, 95.3%) were obtained using the in situ method, by adding 1% PVA to the molasses medium.

### 2.3. High-Performance Liquid Chromatography (HPLC)

The amounts of sugars were quantified by high-performance liquid chromatography (HPLC) on a Shimadzu LC-20A Prominence with refractometric detection using RID10A and a computer controller. Prior to HPLC analysis, all samples and standards were mixed with acetonitrile (ACN) (Kriochrom company, Saint Petersburg, Russia, CAS: 75-05-8, 99.97) (3:1, *v*/*v*), then placed into sealed tubes and centrifuged at 13,000× *g* for 5 min. The SUPELCOSIL™ LC-NH_2_ HPLC column (4.6 × 150 mm) was used, and the mobile phase was ACN: DH_2_O (distilled water) (3:1, *v*/*v*) with a flow rate of 0.5 mL/min. The injection volume was 20 μL, and the column temperature was 40 °C.

### 2.4. Tensile Property Characterization

Tensile tests of various specimens were performed using a PARAM^®^ XLW (PC) Auto Tensile Tester (Labthink, Jinan, China) at a rate of 100 mm min^−1^.

### 2.5. Fourier Transform Infrared (FT-IR) Spectroscopy

BC was freeze-dried and crushed into powder form, mixed with potassium bromide, and pressed into small tablets that were subjected to Fourier transform infrared spectroscopy (FT-IR) using an IRPrestige-21 spectrophotometer (Shimadzu, Kyoto, Japan) in absorption mode. For each sample, 32 scans at 4 cm^−1^ resolution at wave numbers ranging from 4000 to 400 cm^−1^ were collected.

The ratio of crystallinity was determined by the following method:

- The absorbance ratio from 1430 cm^−1^ (A_1430_) and 893 cm^−1^ (A_893_) bands:

Cr.R. = A_1430_/A_893_ (1)

The relative proportion of cellulose I_β_ to I_α_ allomorph could be calculated by integrating the absorption bands near 710 and 750 cm^−1^, and the percentage of I_β_ could be obtained by Equation (2):

(2) $$\%I_{\beta }=\frac{A_{710}}{A_{710}+A_{750}}$$

### 2.6. X-Ray Diffraction (XRD)

X-ray diffraction (XRD) measurement was carried out by an Empyrean x-ray diffractometer (PANalytical, Lelyweg, The Netherlands) using the method described previously [18]. The crystallinity index (CrI) was calculated from the ratio of the height of the 002 peak (I_002_, °2Theta = 22.5°) and the height of the minimum (I_am_) between the 002 and 110 peaks (I_am_, °2Theta = 18°) (Equation (3)).

CrI (%) = [(I_002_ − I_am_)/I_002_] × 100% (3)

### 2.7. Thermogravimetric Analysis (TGA)

Thermogravimetric analysis was performed using a TG 209 F1 Libra thermobalance (Netzsch, Selb, Germany).

### 2.8. Statistical Analysis

All presented data are averages of at least 3 runs of experiments, performed with 3 to 6 replicates of the mean. Standard deviations of the mean were calculated using Microsoft Excel 2013 (Microsoft Corporation, Redmond, WA, USA). The obtained data were statistically analyzed using a Student’s *t*-test, two-sample assuming equal variances. The differences were considered significant at the level of *p* < 0.05.

## 3. Results

### 3.1. BC Production by Agitated Cultivation of K. sucrofermentans B-11267 on a Molasses Medium

#### 3.1.1. Media Comparison

In the present work, we studied BC formation by the *K. sucrofermentans* B-11267 strain under agitated culture conditions in media with different molasses concentrations. We used culture media with molasses concentrations of 50 g/L (M_50_), 70 g/L (M_70_), 90 g/L (M_90_), and 110 g/L (M_110_). The sugar concentration was determined in the culture medium after sterilization at pH values of 6.0 and 4.5 by high-performance liquid chromatography (HPLC) (Table 1).

According to the presented data, no significant changes occurred in the sugar composition during the medium sterilization with an initial pH of 6.0. It contained a significant amount of sucrose and a small amount of glucose. When the medium pH was brought to 4.5, after sterilization at 120 °C for 20 min, there was a decrease observed in the sucrose amount with a simultaneous increase in the glucose, and fructose appeared in the medium. At the same time, the total sugar content remained virtually unchanged.

#### 3.1.2. Effect of Molasses Concentration on BC Production

**Figure 3:**
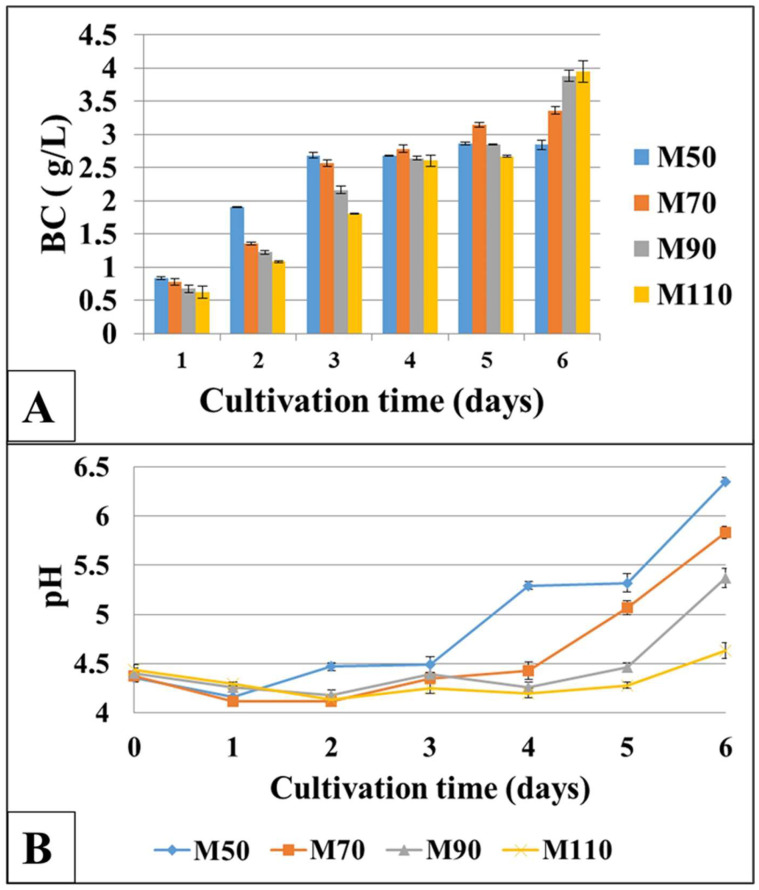
BC production (**A**) and the culture medium pH (**B**) of *K. sucrofermentans* B-11267 at different concentrations of sugar beet molasses.

The dynamics of BC production were investigated in the media with different molasses concentrations with an initial pH of 4.5, since the partial hydrolysis of sucrose to glucose and fructose occurred during sterilization. The results are shown in Figure 3A. The highest amount of BC was formed on day 6 of cultivation in M_90_ and M_110_ media, and showed a total sugar content of 51 and 59 g/L—3.88 ± 0.08 and 3.95 ± 0.16 g/L, respectively. However, during the first three days of cultivation, the highest amount of polysaccharide was found in the M_50_ medium, with a total sugar content of 29 g/L (2.68 ± 0.04 g/L on the third day). On day 5 of cultivation, the maximum BC yield was observed in the M_70_ medium, with a total sugar content of 38 g/L–3.15 g/L. Thus, although the maximum BC yield was observed on day 6 (3.95 ± 0.16 g/L on the M_110_ medium), cultivation for 3 days on the M_50_ medium is a good alternative in economic terms, since the cultivation time is reduced in half in this case; on an industrial scale, this could lead to significant savings in costs.

To assess the vital activity of bacteria, we studied the culture media’s pH dynamics. Figure 3B represents the findings. A slight decrease in pH values was observed on day 1 of cultivation, which might be due to active bacteria growth, the consumption of sugars and the formation of organic acids. On day 2, in all media except the M_50_ medium, there was a further pH decrease. From day 3 to day 6 of cultivation, there was a change in pH values towards the alkaline side. With an increase in the molasses concentration, alkalization occurred to a lesser extent. In the M_90_ and M_110_ media with the maximum amount of sugars, the BC yield increased up to day 6, while the pH values were in the acidic region, reaching values of 5.37 and 4.63, respectively.

**Figure 4:**
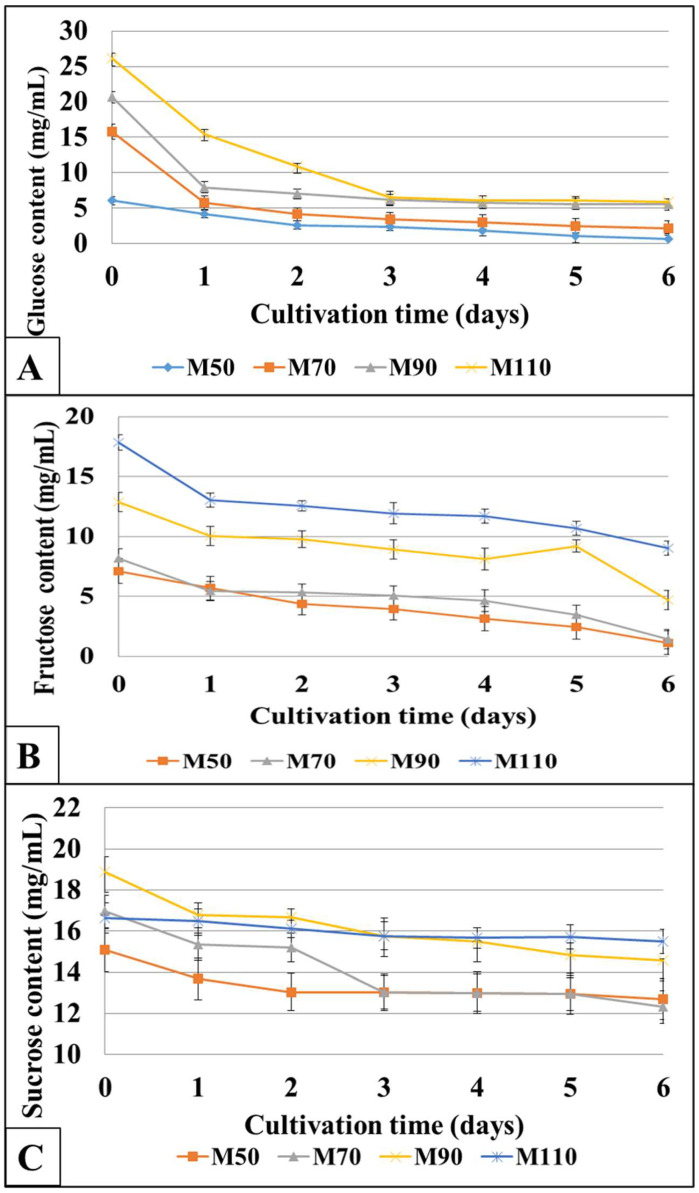
Concentration of glucose (**A**), fructose (**B**), and sucrose (**C**) in the media with different molasses concentrations during the fermentation process (HPLC).

The change in sugar concentration in the *K. sucrofermentans* B-11267 culture medium was also investigated. Our study showed that during the molasses medium’s sterilization with a pH 4.5 without pre-treatment, the partial hydrolysis of sucrose to glucose and fructose also occurred. Figure 4 shows the concentration of sugars (sucrose, glucose, and fructose) in the media with different molasses concentrations during the fermentation process.

The bacterium *K. sucrofermentans* B-11267 consumed glucose at the highest rate in all the studied media (Figure 4A). Moreover, the highest glucose consumption was found within the first 3 days of cultivation. Fructose consumption occurred more gradually throughout the cultivation (Figure 4B). Sucrose consumption occurred less intensively (Figure 4C). That is, by the end of the producer cultivation, a significant amount of sucrose remained in the medium. Thus, in all media, the concentration of sugars decreased steadily with time, indicating that *K. sucrofermentans* B-11267 has the ability to use the carbon sources in these media for BC production. However, in media with different molasses concentrations, glucose was almost completely consumed after 6 days of fermentation (93% in M_50_ and M_70_ media, 72–76% in M_70_ and M_110_ media), while sucrose consumption was only 6–22%. Fructose consumption was 84–86% in M_50_ and M_70_ media, and 24–41% in M_90_ and M_110_ media. Since glucose was always consumed preferably by *K. sucrofermentans* B-11267 [33], it was expected that in sugar beet molasses, after the depletion of glucose, fructose and sucrose would be consumed, respectively. However, in general, it can be concluded that the M_50_ and M_70_ media have advantages in economic terms, since they contain a smaller amount of unused sugars by the end of fermentation, and there is the possibility of reducing the cultivation time, since as early as on day 3 in the M_50_ medium, the BC amount was 2.68 g/L, and in the M_70_ medium, it was −2.5 g/L. Increasing the molasses concentration in the medium to 90–110 g/L significantly increases the amount of unused sugars.

#### 3.1.3. The Morphology of BC and Bacterial Colonies

**Figure 5:**
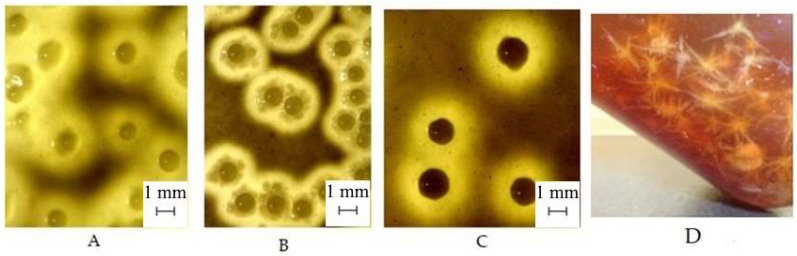
*K. sucrofermentans* B-11267 colonies on agar media with molasses at a concentration of 50 g/L (**A**), 70 g/L (**B**), and 90 g/L (**C**). BC agglomerates formed by *K. sucrofermentans* B-11267 during agitated cultivation in a molasses medium (**D**).

When grown on agar media with molasses at a concentration of 50 g/L, 70 g/L, and 90 g/L, *K. sucrofermentans* B-11267 formed small round colonies that were 1–2 mm in diameter, beige-brown in color, shaped like a ball on a flat base, and surrounded by a transparent halo (Figure 5), which was probably formed under the influence of acetic acid, which is also observed on a medium with chalk [33]. When cultivated under agitated conditions in a liquid medium with molasses at a stirring speed of 250 rpm, the bacteria formed flocculent-shaped BC agglomerates (Figure 5D).

#### 3.1.4. Structural BC Characteristics

##### Fourier Transform Infrared (FTIR) Spectroscopy

**Figure 6:**
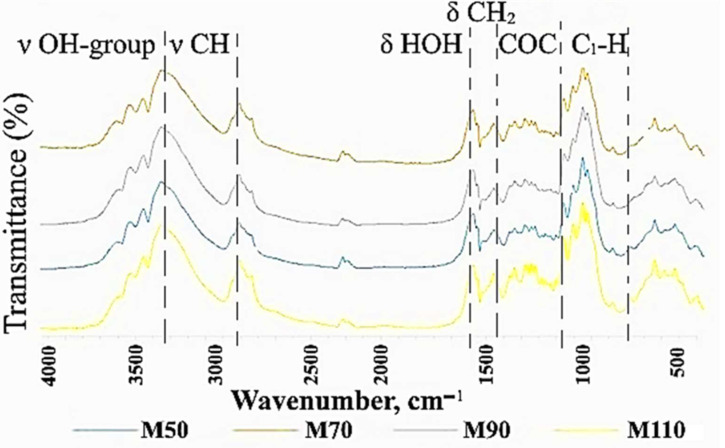
FTIR spectra of BC obtained in media with different molasses concentrations.

The chemical structure of BC was analyzed by FTIR spectroscopy (Figure 6, Table 2). The functional groups of BC obtained from media with different molasses concentrations were almost the same. The characteristic bands of cellulose appeared at 3346, 3355, and 3357 cm^−1^ for the stretching vibration of hydroxyl functional groups (-OH). Due to the presence of highly polar hydroxyl groups in BC, the molecular chains interact by inter- and intra-molecular hydrogen bonds. The absorption bonds at 2897, 2915, 2920, and 2921 cm^−1^ are characteristic of the symmetric and anti-symmetric C-H stretching of CH_2_ and CH_3_ functional groups. The absorption bonds at 1653 and 1655 cm^−1^ were attributed to the H-O-H bending of absorbed water. The bonds at 1428 and 1429 cm^−1^ could be associated with CH_2_ symmetric bending vibrations. The peaks at 1371, 1374, and 1377 cm^−1^ represented the S ring stretching (CH_2_ rocking at C_6_). The peaks at 1161 and 1163 cm^−1^, and 1055, 1059, and 1060 cm^−1^ were attributed to the asymmetric stretching vibration of glycosidic C-O-C and the C-O symmetric stretching vibrations of the pyranose ring. The β-glucosidic bonds across glucose rings resulted in the peaks at 895–897 cm^−1^. The characteristic bands at 1430, 1163, and 896 cm^−1^ indicate cellulose I as the major component. Cellulose I is the form of cellulose synthesized by bacteria, and is composed of parallel β-1,4 glucan chains. There are two different crystalline phases in cellulose I, called I_α_ and I_β_. BC displays the properties of small crystallite size, high crystallinity, and high cellulose I_α_ content. The crystallinity index and the content of the α and β phase of cellulose in our samples were determined by a method based on the peak intensity ratio (Table 3). The results showed that BC obtained in media with different molasses concentrations generally have an identical crystallinity index. However, with an increase in the molasses concentration, a slight decrease in the peak intensity ratio was observed.

##### X-Ray Diffraction (XRD)

**Figure 7:**
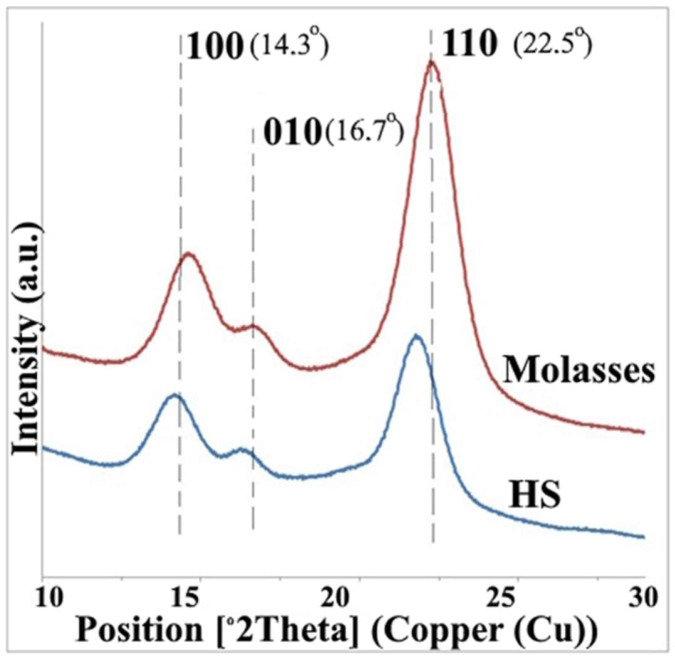
XRD patterns of BC obtained from HS medium and molasses.

In order to analyze the crystallinity of BC produced from molasses and HS culture media, X-ray diffraction was used. The obtained X-ray patterns demonstrate cellulose with the same chemical structure but different crystallinity degrees (Figure 7). The diffraction diagrams produced by all BC samples show three main peaks at °2Theta 14.3°, 16.7°, and 22.5°, corresponding to crystallographic planes of (100), (010), and (110), respectively. The findings showed that on the medium with molasses, the BC crystallinity degree (83%) was higher than that in the standard HS medium (79%).

### 3.2. Production of Tubular Materials by Culturing K. sucrofermentans B-11267 on a Molasses Medium

#### 3.2.1. Production of BC and BC/PVA Tubular Structures

**Figure 8:**
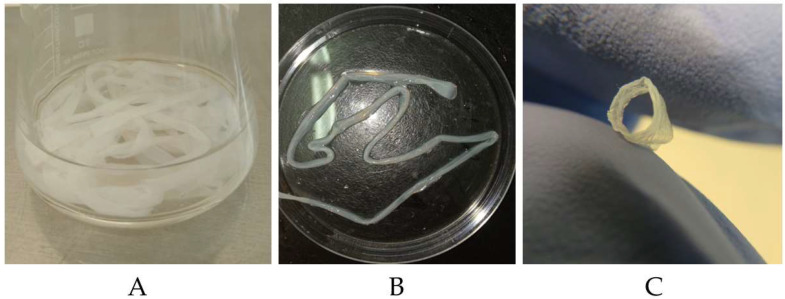
Tubular structures of native BC (**A**) and BC/PVA composite (**B**) obtained by culturing *K. sucrofermentans* B-11267 in a molasses medium after purification. Cross-section of tubular structure (**C**).

**Figure 9:**
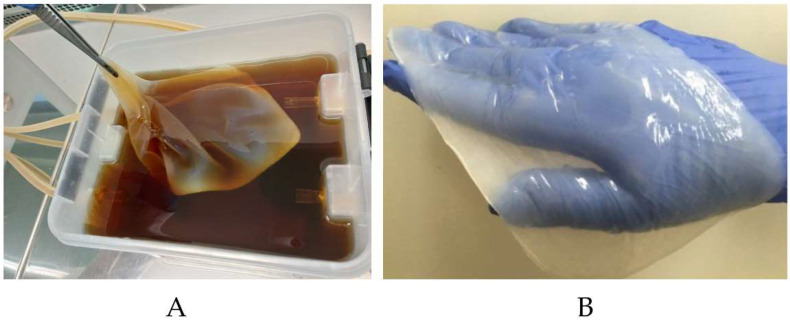
BC gel film formed on the surface of the medium in the container (**A**) and after purification (**B**).

In our study, tubular BC structures were produced by culturing *K. sucrofermentans* B-11267 in a bioreactor, which represents a container with two 200 cm silicone tubes, 5 mm in their internal diameter (Figure 2), using molasses at a concentration of 50 g/L. The volume of culture liquid in the bioreactor as a whole was 1500 mL, of which 1422 mL was in the container and about 78 mL was in two silicone tubes. The *K. sucrofermentans* B-11267 was cultured for 7 days, with double mixing of the nutrient medium in each tube for 10 min on days 1 and 2. As a result of the cultivation, two tubular structures were obtained, each 200 cm long (Figure 8A). In addition, a BC gel film formed on the surface of the nutrient medium in the container (Figure 9). The dry BC yield was 0.9 g/L (0.67 g/L for a film and 0.23 g/L for tubular structures). The resulting structures were processed to remove cells and components of the nutrient medium and sterilized. Composite tubular structures based on BC and PVA were also obtained by the in situ method by adding 1% PVA to the molasses medium. Figure 8B demonstrates the resulting structures after purification.

#### 3.2.2. Characteristics of BC and BC/PVA Tubes

##### Mechanical Properties

We investigated the mechanical properties of wet tubular structures based on BC and BC/PVA by determining their tensile strength. Tensile testing is a commonly accepted method for the mechanical characterization of vascular structures in the literature. The results are presented in Table 4. The BC/PVA composite thickness was 22% greater than that of the native BC tubular structures. The percentage of tensile elongation of the materials for BC and the composite did not differ significantly. Tubular BC/PVA composites obtained by the in situ method during *K. sucrofermentans* B-11267 cultivation on a medium with molasses and PVA turned out to be stronger. The tensile strength of the composite was almost two times higher than that of the native BC tubular structures.

##### FTIR Spectroscopy

**Figure 10:**
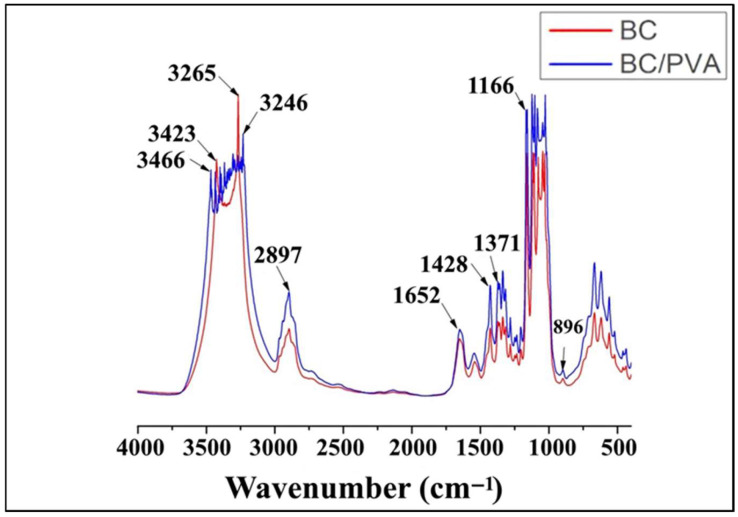
FTIR spectra of BC and BC/PVA composite.

The structure of the tubular materials was characterized by FTIR spectroscopy. The spectra of BC/PVA composite were similar to those registered for pure BC (Figure 10). However, the hydroxyl band in the range of 3000–3600 cm^−1^ was wider in the spectra of composite, which confirmed the formation of intermolecular interactions between hydroxyl groups of PVA and BC. The characteristic bands of cellulose appeared at 3423 and 3265 cm^−1^ for the stretching vibration of hydroxyl functional groups (-OH). Due to the presence of highly polar hydroxyl groups in BC, the molecular chains interact by inter- and intra-molecular hydrogen bonds. The absorption bond at 2897 cm^−1^ is characteristic of the symmetric and anti-symmetric C-H stretching of CH_2_ and CH_3_ functional groups. The absorption bond at 1652 cm^−1^ was attributed to the H-O-H bending of absorbed water. The bonds at 1428 cm^−1^ could be associated with CH_2_ symmetric bending vibrations. The peaks at 1371 cm^−1^ represented the S ring stretching (CH_2_ rocking at C_6_). The peak at 1166 cm^−1^ was attributed to the asymmetric stretching vibration of glycosidic C-O-C of the pyranose ring. The β-glucosidic linkages across glucose rings result in the peak at 896 cm^−1^.

##### XRD Analysis

**Figure 11:**
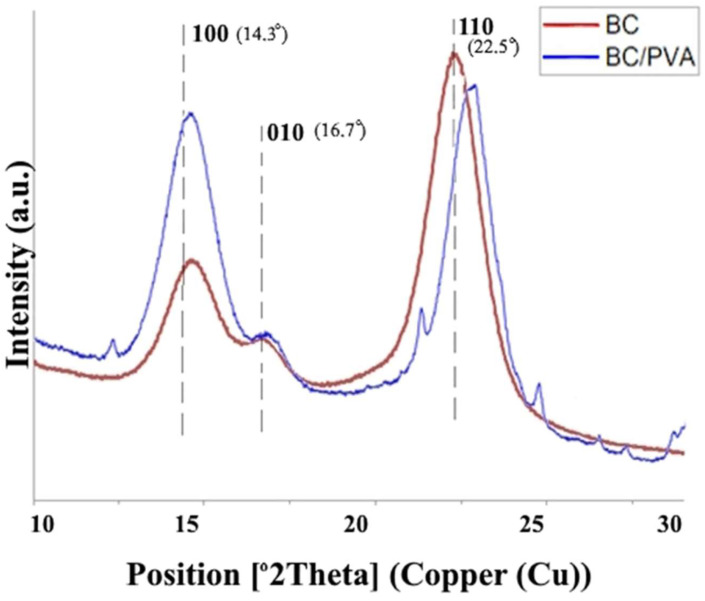
XRD patterns of BC and BC/PVA tubes obtained from molasses.

X-ray diffraction was used to evaluate the crystal structure of BC tubes and BC/PVA composite tubes. The obtained X-ray patterns show the cellulose with the same chemical structure but different crystallinity degrees (Figure 11). The findings showed that, on the molasses medium, the crystallinity degree of BC tubular structures (83%) was higher than that of BC/PVA composite (64%).

##### Thermal Properties

**Figure 12:**
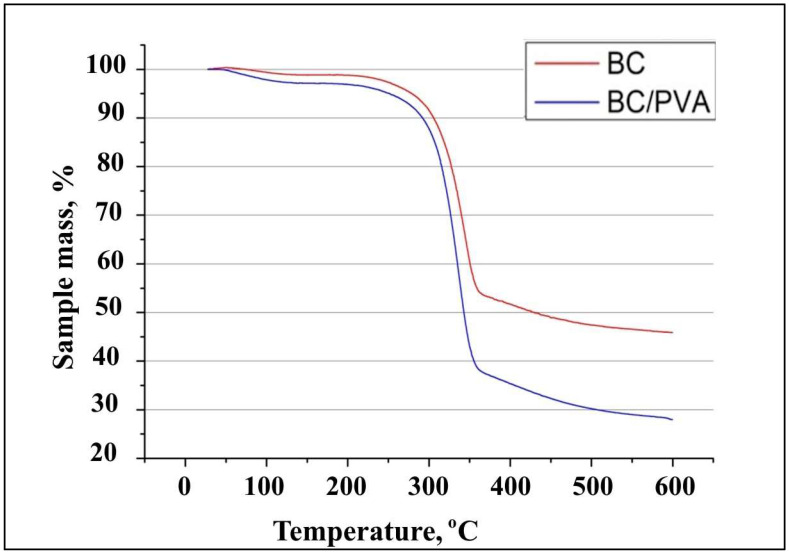
TG curves of tubular structures from native BC and BC modified with PVA by the in situ method in a molasses medium.

The thermal stability and degradation profile of BC tubular structures and a BC/PVA composite were assessed to evaluate their potential use in high-temperature applications, such as thermal processing, by thermogravimetric (TG) analysis. Figure 12 shows the TG curves of tubular structures from native BC and BC modified with PVA by the in situ method in a molasses medium. The results demonstrated the weight loss of the samples began at 55 °C, and it did not exceed 5% for the native BC sample up to 280 °C and the BC/PVA composite up to 250 °C, which was due to the evaporation of water bound with BC. The native BC sample retained thermal stability up to 305 °C, and the modified PVA sample retained thermal stability up to 290 °C. With a further increase in temperature, the materials were destroyed. At the same time, the native BC sample was more thermally stable, since at 340 °C its weight loss was 30%, while the weight loss of the modified BC sample was 47%.

## 4. Discussion

In the present work, we studied the BC production and tubular BC structures under specific cultivation conditions on a molasses medium. Molasses is a by-product of processing sugar cane and sugar beet in sugar production [34]. The cost of molasses is only 1/250 of that of fructose [19]. Machado et al. (2018) showed that the use of sugarcane molasses contributes to a significant reduction in the cost of media (up to 20.06%) [20]. Additionally, Rouhi et al. (2023) demonstrated that the BC production cost was reduced by about 94% using a cost-effective medium containing molasses and corn steep liquor [11]. The present work highlighted the use of beet molasses, which allowed a reduction in the medium cost by about 98% compared to the standard HS medium. Beet molasses contains on average about 80% dry matter and 20% water, a significant part of which is in a bound state due to hydration in a solution of colloids, sucrose molecules, and mineral ions. The amount of sucrose in beet molasses varies from 48 to 62% of its weight. The composition of molasses also includes organic acids and their salts, amino acids, betaine, mineral compounds, and vitamins [35].

Our studies showed that, when sterilizing a medium with molasses at pH 4.5 without pre-treatment, the partial hydrolysis of sucrose to glucose and fructose occurred. Previously, a number of researchers showed a similar process to occur with a more severe thermal acid treatment, in which molasses is pre-diluted with distilled water before centrifugation. Then, the supernatant pH is brought to 3.0 with 4 N H_2_SO_4_ and heated to 120 °C for 20 min followed by being kept at a room temperature [19,36,37,38,39]. After that, it is used to prepare nutrient media with subsequent sterilization.

Although molasses has been used for BC production for a long time, as evidenced by the works by Bae and Shoda (2004) [19], Keshk et al. (2006) [40], Cakar et al. (2014) [38], Tyagi and Suresh (2016) [36], and Machado et al. (2018) [20], studies devoted to various aspects of its application are continuing, are of great interest of researchers and have been presented over the past five years in articles by Salari et al. (2019) [41], Abol-Fotouh et al. (2020) [39], Revin et al. (2021) [18], Rouhi et al. (2023) [11], and Khanchezar et al. (2024) [15].

Several authors have already described the production of BC in molasses media using various bacterial strains, including *G. xylinus* ATCC 10245 [40], *G. xylinus* FC01 [38], *A. xylinum* BPR2001 [15], *G. xylinus* PTCC 1734 [41], *G. intermedius* SNT-1 [36], *A. xylinum* subsp. *sucrofermentans* BRP2001 [19], *K. rhaeticus* [20], *K. saccharivorans* MD1 [39], and *K. sucrofermentans* H-110 [18]. However, most works on obtaining BC were carried out by culturing producers under static conditions [11,41].

Considering that the studies devoted to obtaining BC from such a cheap and renewable source of valuable nutrients as molasses are ongoing and of great interest to researchers, as well as the features of BC formation by different strains, and the fact that most of the works on obtaining BC on molasses were carried out during the cultivation of producers under static conditions, the aim of the present work was to study BC production under agitated culture conditions of culturing the strain *K. sucrofermentans* B-11267 on media with different concentrations of molasses. The research showed the maximum BC accumulation to be 3.95 g/L on day 6 of cultivation in a medium, the molasses concentration being 110 g/L, and a total sugar content 59 g/L. However, on day 3 of cultivation, the greatest amount of polysaccharide (2.68 g/L) was formed in a medium with a molasses concentration of 50 g/L and a total sugar content of 29 g/L. It might be related to the fact that the high content of sugars and other elements contained in media with molasses in concentrations above 50 g/L suppress the production of cellulose at the initial stages of cultivation. In all our experiments, the pH values were in the range of pH 4.0–6.0, which, according to most literature data, is optimal for BC biosynthesis [1,17,42]. The pH value of the culture medium plays a critical role in both cell growth and BC production. At high glucose concentrations, the excess glucose that is not used for cellulose synthesis is oxidized to gluconic acid. The accumulation of gluconic acid drastically decreases the pH of culture medium and inhibits BC production. It can be related to the BC production decrease in the HS medium containing glucose as the sole carbon source. On the other hand, sugar beet molasses contains a lower amount of glucose, leading to the lower gluconic acid formation, and consequently, more BC production in these media.

The dynamics of sugar consumption during the cultivation were studied by HPLC. *K. sucrofermentans* B-11267 was shown to consume glucose more intensively, while fructose and sucrose consumed to a lesser extent. Glucose is considered the main source for BC production. Since it is a precursor for cellulose synthesis, all compounds that can be converted into glucose are capable of forming BC. Structural isomers of glucose (e.g., fructose) and glucose precursors (e.g., glycerol and mannitol) are easily transformed into glucose, and enter into cycles of glucose phosphorylation, glucose-6-phosphate isomerization, and UDP-glucose synthesis with subsequent BC production. In media with a high concentration of molasses, a lot of unconsumed sugars remained.

The chemical structure of BC was analyzed by FTIR spectroscopy. The functional groups of BC obtained from media with different molasses concentrations were almost the same. The study showed that BC consists of pure cellulose and does not contain any other components. X-ray diffraction (XRD) measurement demonstrated that, on the medium with molasses, the BC crystallinity degree (83%) was higher than that in the standard HS medium (79%). This is consistent with the previously obtained results for the cultivation of *K. sucrofermentans* B-11267 under static conditions [18]. In addition, our previous studies showed that, during the dynamic cultivation of *K. sucrofermentans* B-11267 on native food industry waste with high acidity, the maximum BC crystallinity degree was found when using the stillage (82.3%), which was slightly higher than that of BC from the HS medium, and significantly higher than that of BC from whey (50.2%) [17]. Thus, the findings indicate that *K. sucrofermentans* B-11267, when cultivated under agitated culture conditions with such a cheap waste medium from the sugar industry as molasses, forms a significant amount of BC with a higher crystallinity degree than on a standard and more expensive HS medium.

BC production, in contrast to other microbial exopolysaccharides (xanthan, levan, alginate, etc.), is not only carried out by cultivation under agitated culture conditions, but also under static conditions. In this case, the BC gel film forms on the medium surface at the liquid–air interface. Bacterial EPS play an important role in bacterial aggregation, adhesion, and biofilm formation [43,44]. BC is an architectural and functional component of biofilms of many bacteria [45]. The biofilm matrix protects cells against various external agents and from drying out, and promotes horizontal gene transfer and intercellular interactions [1]. BC film on the medium surface helps to create aerobic conditions for bacteria.

Another interesting form of the non-trivial self-organizing behavior of microorganisms under specific cultivation conditions is the formation of tubular structures. In case of the formation of tubular structures, bacterial cells attach to the inner surface of silicone tubes forming the biofilms, in which the main component of the matrix is cellulose. The BC’s ability to take a certain shape during biosynthesis makes it possible to produce tubular structures of different diameters and lengths, depending on the required application.

Various BC tubular structures obtained in different types of fermenters and their potential for creating artificial blood vessels are reported [26,30,32,46,47,48]. The review by Roberts et al. (2023) summarized the strategies used to fabricate tubular BC structures, and reported various types of fermenters, each with different advantages and disadvantages, yielding BC tubes with varying properties [32]. Authors reported four types of methods to produce tubular structures: pellicle fermentation methods, cyclical fermentation methods, static fermentation methods, and dynamic fermentation methods [32]. The film-forming method involves obtaining the BC initially as a film and then shaping it into a tube. Other methods use tube-shaped fermenters made of glass or oxygen-permeable materials positioned vertically or horizontally. Fermentation without gas exchange is considered static, and that with continuous gas exchange is considered dynamic. The present work proposes an original design of a simple bioreactor for obtaining tubular structures of any length in silicone tubes, which unlike glass tubes are permeable to oxygen. Furthermore, this method, in addition to tubular structures, enables us to simultaneously obtain a BC gel film on the nutrient medium surface. Thus, the method we propose has such advantages as its simplicity, the use of silicone tubes permeable to oxygen to solve the aeration problems, and the possibility to obtain tubular structures of great length with the simultaneous production of a bacterial cellulose gel film in a container.

All the known tubular BC structures were obtained in the media, which contains expensive components, e.g., glucose, fructose, peptone, yeast extract. In our study, for the first time, we developed tubular materials based on BC that were obtained on a molasses medium. In addition, a tubular BC-based biocomposite with improved mechanical properties was obtained for the first time by combining the cultivation on the molasses medium with the in situ chemical modification by PVA. The synthetic polymer was chosen due to its non-toxicity, hemocompatibility, water solubility, and compatibility with BC due to the presence of hydroxyl groups [49]. For all types of tissue-engineered vessels, their mechanical characteristics are of utmost importance to predict whether the structure is capable of withstanding the mechanical load of the intended application [50]. Tubular BC/PVA composites obtained by the in situ method during *K. sucrofermentans* B-11267 cultivation on a medium with molasses and PVA turned out to be stronger. The tensile strength of the composite was almost two times higher than that of the native BC tube. Previously, Długa et al. (2021) showed the Young’s modulus of BC/PVA composites obtained in situ to be similar or lower than those of BC, while the stress and strain at break were higher [49]. The authors explained this fact by the highest flexibility and resistance to tearing due to PVA in the BC fibers. In another study, Tang et al. (2015) obtained tubular BC/PVA composites with a significantly greater tensile strength of 0.45 MPa compared to 0.1 MPa of unmodified BC by using a thermally induced phase separation method [31]. The present work demonstrated that the FTIR spectra of BC/PVA composite were similar to those registered for pure BC. However, the hydroxyl band in the range of 3000–3600 cm^−1^ was wider in the spectra of composite (Figure 9), which confirmed the formation of intermolecular interactions between hydroxyl groups of PVA and BC [49]. XRD measurement demonstrated that, on the molasses medium, the crystallinity degree of BC tubular structures (83%) was higher than that of the BC/PVA composite (64%). Similar data have been presented in a number of other studies. For example, Długa et al. (2021) demonstrated that the crystallinity degree of BC/PVA composites was lower than that of BC, and decreased with increasing PVA content (1%, 2%, and 4%), which, according to the authors, indicates that PVA complicates the ordering of the cellulose chain in the composites and, as a result, reduces the duration of the crystalline phase [49]. In the field of biomedicine, thermal stability is a key property that enables to be thermally processed, and used to obtain polymer composites [51,52]. Thus, the thermal stability and degradation profile of BC tubular structures and the BC/PVA composite were assessed to evaluate their potential use in high-temperature applications such as thermal processing by TGA. The results demonstrated that the native BC sample was more thermally stable. A number of researchers point out that the thermal stability of any material is intrinsically dependent on its crystallinity, since higher crystallinity means a stronger polymer chain; therefore, the breakage between the chains will be difficult [52].

## 5. Conclusions

The present work, for the first time, performed a study of cost-effective BC production and tubular BC structures by the *K. sucrofermentans* B-11267 strain under specific cultivation conditions on a molasses medium. As far as we know, molasses has not been used to obtain tubular BC structures, although it is widely used for BC production. Molasses does not only reduce the medium cost by about 98% compared to the standard HS medium, but also promotes the formation of a biopolymer with high mechanical strength and high crystallinity. An original design of a simple bioreactor, which represents a container with two silicone tubes, was used to fabricate two tubular BC structures 200 cm long. The method we propose has such advantages as its simplicity, the use of silicone tubes permeable to oxygen, and the possibility to obtain tubular structures of great length with the simultaneous production of a bacterial cellulose gel film in a container. In addition, a tubular BC-based biocomposite with improved mechanical properties was obtained by combining the cultivation on the molasses medium with in situ chemical modification by polyvinyl alcohol. The tensile strength of the composite was almost two times higher than that of the native BC tube. The BC tubular structures demonstrated great potential for their application in biomedicine as artificial blood vessels and conduits for nerve regeneration.

## Figures and Tables

**Table 1:** Amount of sugars in the medium with different molasses concentrations after sterilization at pH values of 6.0 and 4.5 (HPLC).

Medium	Molasses Concentration, g/L	pH	Concentration of Sugars After Sterilization, g/L			
			Sucrose	Glucose	Fructose	Total Sugars
M_50_	50	6.0	25.38 ± 0.50	4.22 ± 0.44	_	29.60 ± 0.4
		4.5	15.13 ± 0.61	6.02 ± 0.71	8.04 ± 0.25	29.19 ± 0.4
M_70_	70	6.0	35.2 ± 0.39	4.75 ± 0.25	_	39.95 ± 1.1
		4.5	16.68 ± 0.69	13.89 ± 0.83	7.56 ± 0.25	38.13 ± 1.2
M_90_	90	6.0	43.0 ± 0.69	7.71 ± 0.42	_	50.71 ± 0.5
		4.5	18.4 ± 0.77	20.76 ± 0.76	11.84 ± 0.19	51.0 ± 0.5
M_110_	110	6.0	55.44 ± 0.82	8.55 ± 0.33	_	63.99 ± 3.2
		4.5	16.65 ± 0.40	25.6 ± 0.55	17.05 ± 15	59.3 ± 2.5

**Table 2:** Results of FTIR spectra of BC obtained in media with different molasses concentrations.

Characteristics Peaks Position	Media			
	M_50_	M_70_	M_90_	M_110_
ν OH-group stretching vibration	3346	3346	3355	3357
ν CH stretching vibration	2920	2921	2920	2915
δ HOH absorbed	1653	1653	1653	1655
δ CH_2_ vibration	1429	1429	1429	1428
δ in plane OH bending CH_2_OH	1371	1371	1374	1377
ν asymmetric C-O bridge stretching anhydroglucose ring C-OR stretching C-O-C pyranose ring skeletal vibration	1163	1161	1163	1163
	1059	1059	1060	1055
C_1_-H rock vibration	896	897	896	895

**Table 3:** Crystallinity index and content of α and β phases in BC obtained in media with different molasses concentrations.

Medium	I_α_, %	I_β_, %	Crystallinity Index
M_50_	43.9 ± 0.90	56.1 ± 0.90	1.60 ± 0.06
M_70_	42.1 ± 1.05	57.9 ± 1.05	1.57 ± 0.05
M_90_	41.9 ± 1.4	58.1 ± 1.4	1.54 ± 0.09
M_110_	40.5 ± 1.3	59.5 ± 1.3	1.48 ± 0.03

**Table 4:** Mechanical properties of BC and BC/PVA tubes.

Characteristics	BC	BC/PVA
Thickness, µm	141.2 ± 11.9	180.5 ± 25.0
Tensile strength (MPa)	1.38 ± 0.14	2.41 ± 0.23
Elongation at break (%)	30.6 ± 1.4	30.1 ± 1.6

## Data Availability

Sequence data are available from GenBank, NCBI.
